# Supplementary material for: RADIP technology comprehensively identifies H3K27me3-associated RNA–chromatin interactions
Source: Nucleic Acids Res. 2024 Nov 18;52(22):e104. doi: 10.1093/nar/gkae1054 (PMC11662664; doi:10.1093/nar/gkae1054)
Supplement: gkae1054_Supplemental_Files [file gkae1054_supplemental_files.zip › Resubmit Shu RADIP_NAR_format_Supplementary Figures_20241011.pdf]

Figure S1.

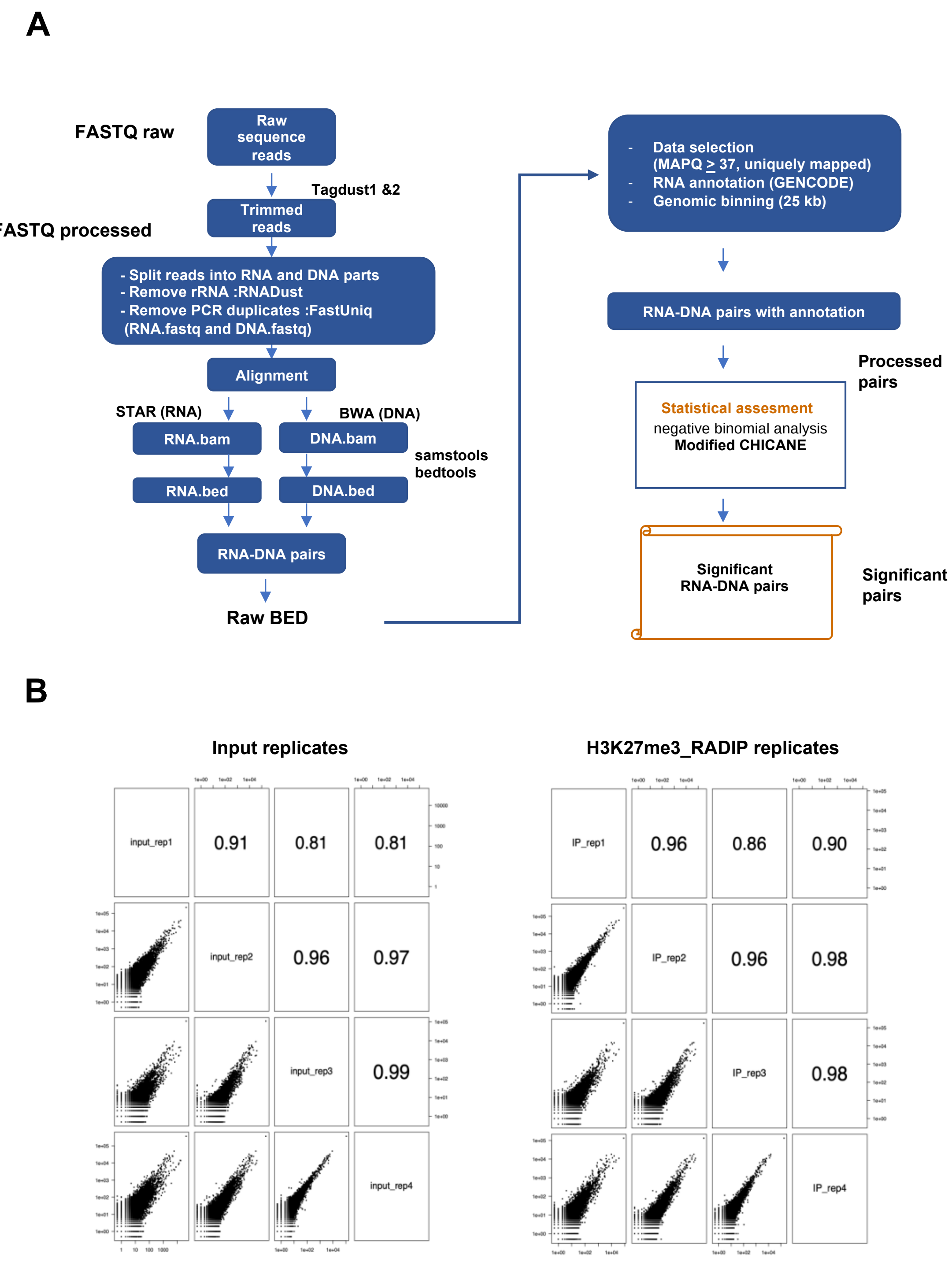
A
FASTQ raw
Raw sequence reads
Tagdust1 &2
Trimmed reads
FASTQ processed
- Split reads into RNA and DNA parts
- Remove rRNA :RNADust
- Remove PCR duplicates :FastUniq
(RNA.fastq and DNA.fastq)
Alignment
STAR (RNA)
BWA (DNA)
RNA.bam
DNA.bam
samstools
bedtools
RNA.bed
DNA.bed
RNA-DNA pairs
Raw BED
- Data selection (MAPQ ≥ 37, uniquely mapped)
- RNA annotation (GENCODE)
- Genomic binning (25 kb)
RNA-DNA pairs with annotation
Processed pairs
Statistical assesment
negative binomial analysis
Modified CHICANE
Significant RNA-DNA pairs
Significant pairs
B
Input replicates
input_rep1
0.91
0.81
0.81
input_rep2
0.96
0.97
input_rep3
0.99
input_rep4
H3K27me3_RADIP replicates
IP_rep1
0.96
0.86
0.90
IP_rep2
0.96
0.98
IP_rep3
0.98
IP_rep4

Figure S1. Data-processing flowchart and high reproducibility of H3K27me3-RADIP technology. (**A**) Starting with the raw sequence reads, the adaptor sequences were trimmed off and the flanking sequences were split into the respective mate pairs of RNA and DNA reads on the basis of the orientation of the internal bridge adaptor. After the rRNA had been removed, data filtering and genomic alignment were performed. Only uniquely mapped RNA–DNA pairs were stored and annotated. The genome-wide RNA–DNA interactome was calculated in a matrix by evaluating captured RNA–DNA pairs between RNA species and genomic bins of 25 kb, with reporting of the processed pairs of all chromatin-associated RNAs on the binned genome. RNA–DNA pairs were processed by background removal, and significant pairs were output for downstream analysis. (**B**) Following these methodologies, RADIP technology exhibited high reproducibility among four Input replicates and four H3K27me3-RADIP replicates. BAW, Burrows–Wheeler Aligner; MAPQ, mapping quality.

Figure S2.

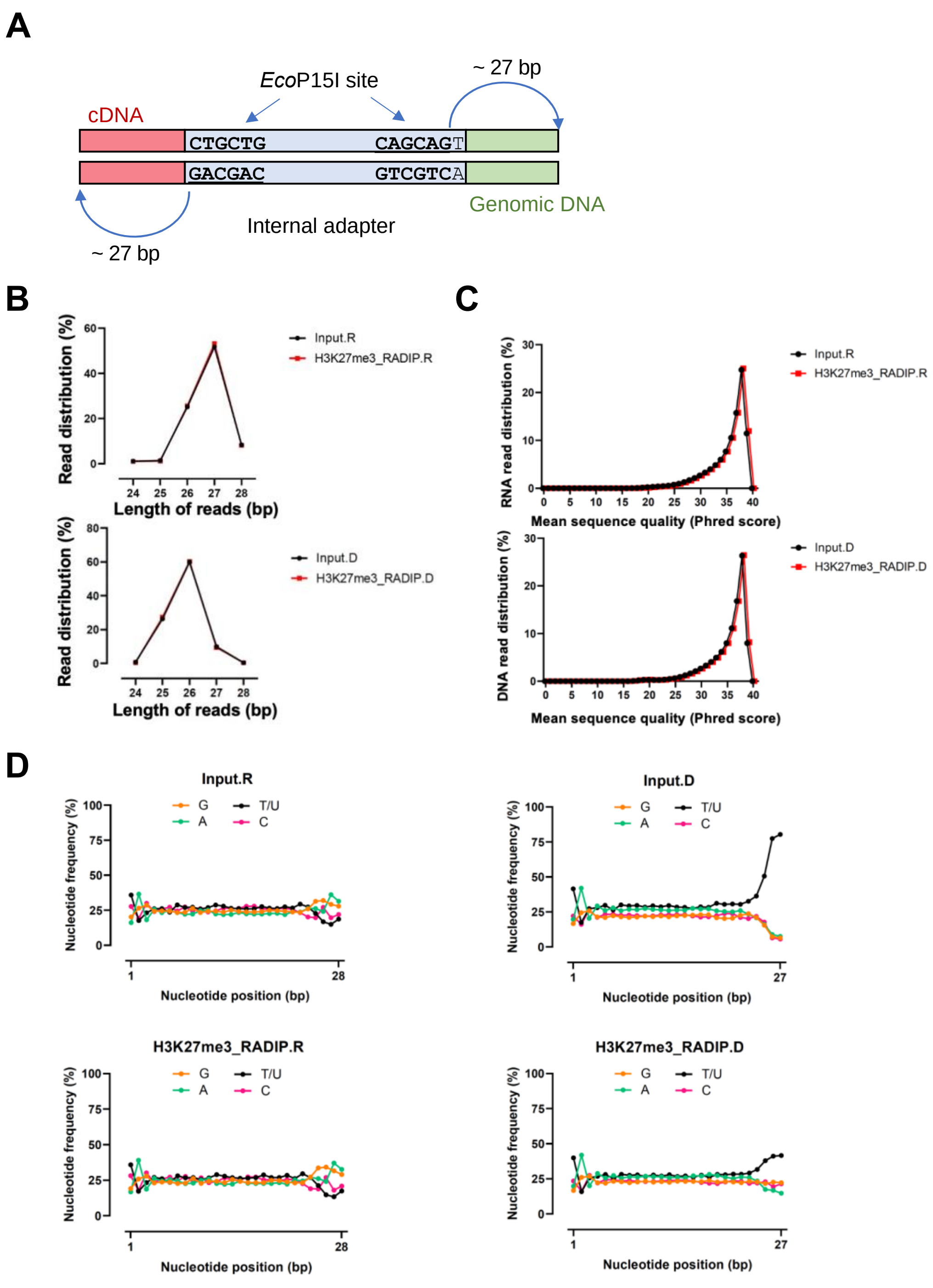

Figure S2. H3K27me3-RADIP data quality and features.

(**A**) Features of RNA–adaptor–DNA chimeric molecules. (**B**) Summary of lengths of RNA and DNA tags of Input and H3K27me3-RADIP samples. (**C**) Report of the base-call accuracy of RNA and DNA tags of Input and H3K27me3-RADIP samples. (**D**) Nucleotide frequencies of RNA (left) and DNA (right) tags of Input and H3K27me3-RADIP samples, showing that the distributions of nucleotides in the RNA and DNA tags were uniform, with the exception of the overhang of the T and Ecop151 recognition motif on the two ends of the DNA tags.

Figure S3.

A

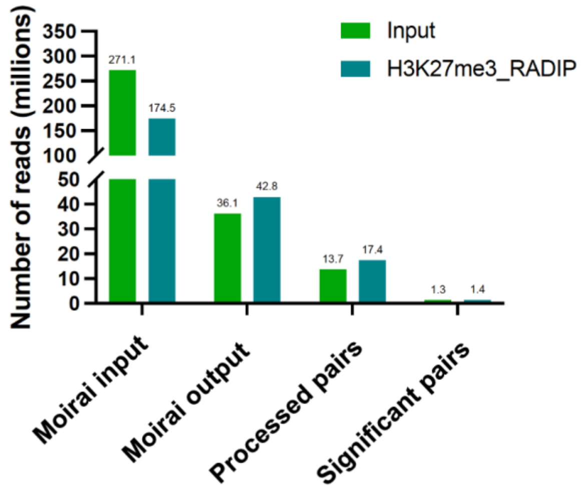

B

Processed pairs

Before (RNA-DNA pairs)	*Cis* reads number	*Trans* reads number
Input	4,269,577	9,429,349
H3K27me3_RADIP	4,684,311	12,675,142

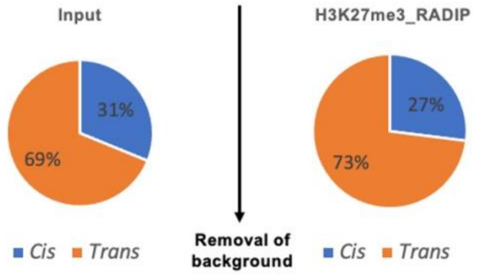

Significant pairs

After (caRNA-DNA pairs)	*Cis* reads number	*Trans* reads number
Input	1,300,602	4,505
H3K27me3_RADIP	1,409,822	5,313

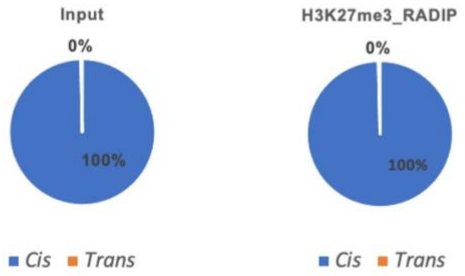

Figure S3. Details of background removal.

(**A**) Summary of sequenced Input and H3K27me3-RADIP libraries. Numbers above the bars represent the replicate-combined numbers of raw reads or the mate pairs remaining after the individual data-processing steps. (**B**) After removal of the background, many of the *trans* interactions were eliminated owing to their low frequency. The proportions and actual numbers of *cis* and *trans* RNA–DNA interaction pairs are shown.

Figure S4.

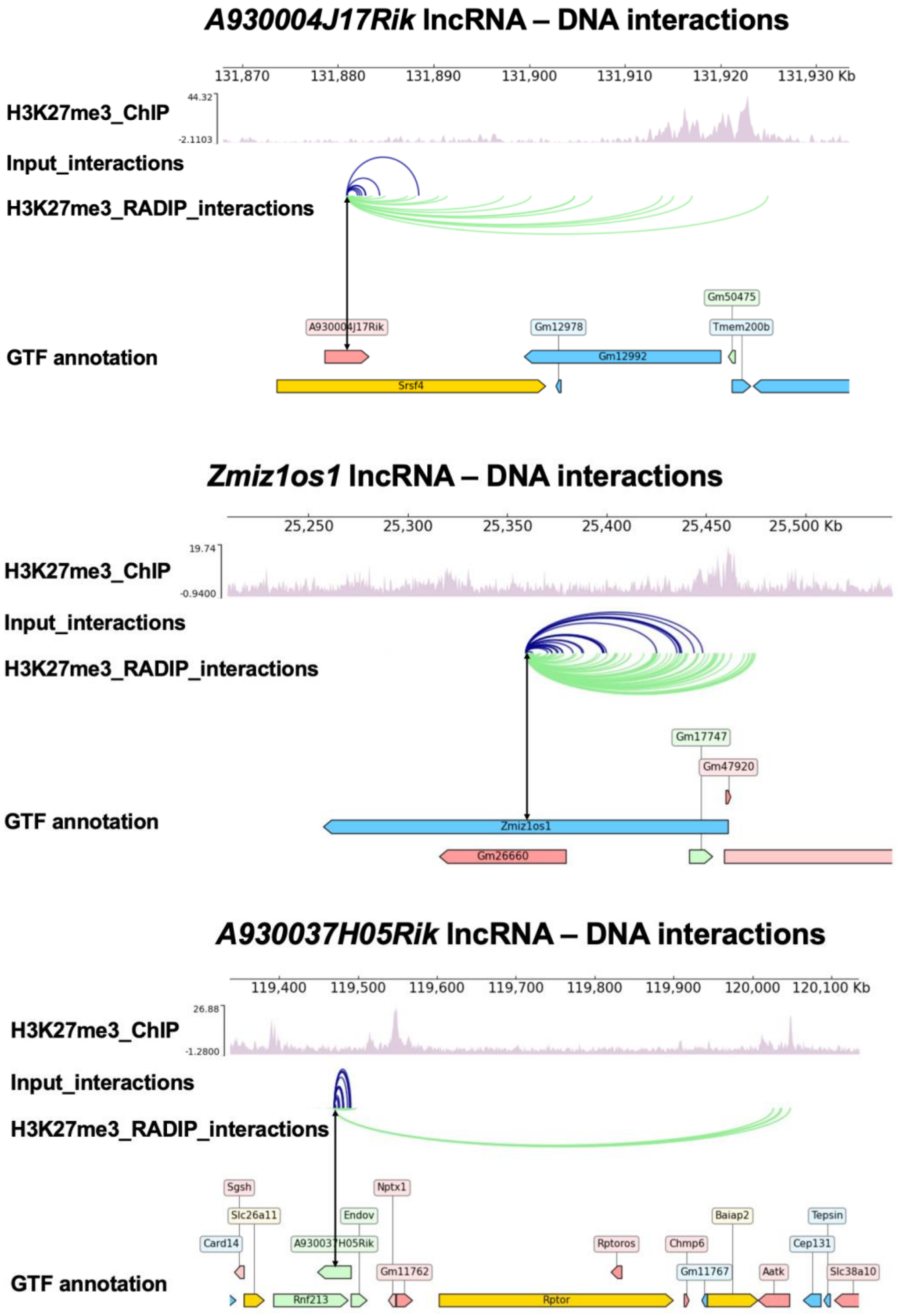

Figure S4. H3K27me3-RADIP samples are enriched with lncRNAs interacting with H3K27me3 ChIP-seq peak DNA regions.

A930004J17Rik lncRNA, *Zmiz1os1* lncRNA, and *A930037H05Rik* lncRNA were used as examples to show that the number of lncRNA–DNA interaction pairs was much greater in the H3K27me3-RADIP samples than in the Input samples. In addition, the target DNA tags of these lncRNAs in the H3K27me3-RADIP samples were in strong agreement with the H3K27me3 ChIP peaks.

Figure S5.

A

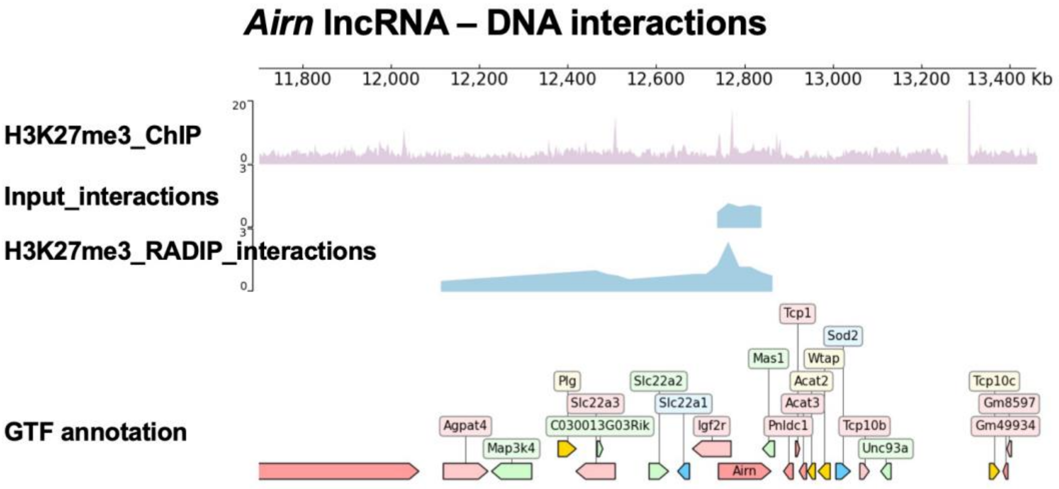

B

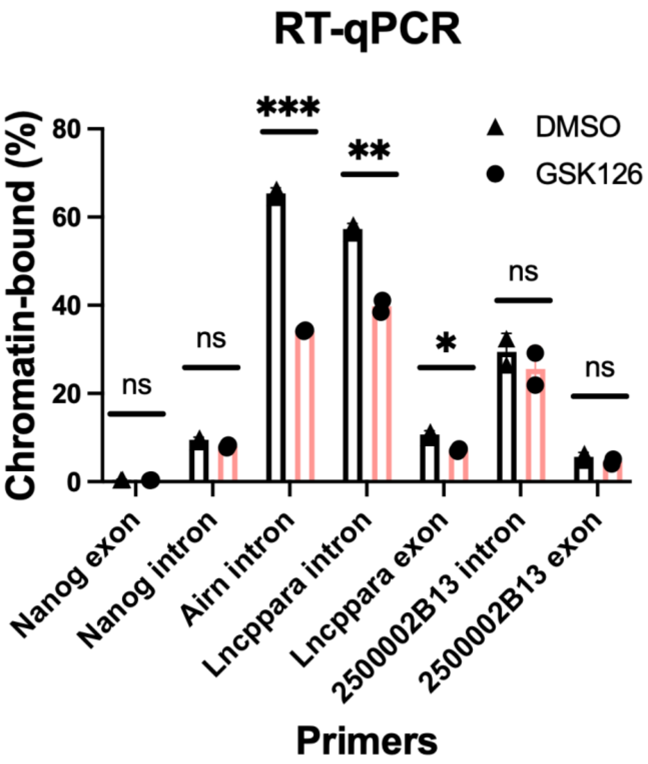

Figure S5. Certain RNA–PRC2 complexes are physically attached to chromatin.

(**A**) H3K27me3-RADIP samples showed relatively high chromatin access of *Airn* lncRNA compared with Input samples, especially in the H3K27me3 ChIP-seq peak DNA regions. (**B**) The levels of *Airn* intron and *Lncppara* intron and exon in the chromatin-associated fraction were significantly reduced following treatment with GSK126. In contrast, the levels of *Nanog* intron and the intron and exon of *2500002B13* lncRNA, used as negative controls, remained unchanged in the chromatin-associated fraction. The source data and details for these results have been added to Supplementary Table S3. (Independent t-test; P-value < 0.05: *; P-value < 0.01: **; P-value < 0.001: ***)

Figure S6.

A

chr.6

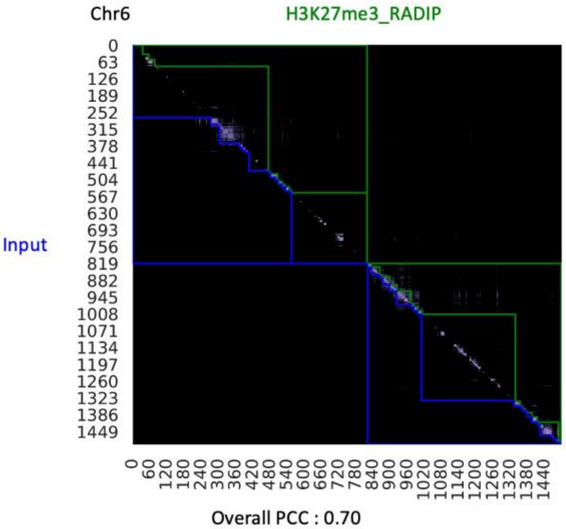

Input H3K27me3_RADIP

```
[ 1 35] is a split TAD from [  1 272]
[ 81 473] is a merged TAD from [407 472]
[474 477] is a matched TAD with [473 476]
[478 480] is a merged TAD from [477 477]
[481 484] is a split TAD from [482 496]
[485 506] is a merged TAD from [499 506]
[507 511] is a merged TAD from [507 509]
[512 522] is a split TAD from [510 522]
[523 531] is a split TAD from [523 533]
[532 532] is a split TAD from [523 533]
[533 546] is a matched TAD with [534 547]
[547 555] is a merged TAD from [548 552]
[556 819] is a matched TAD with [555 820]
[820 833] is a matched TAD with [821 833]
[834 863] is a merged TAD from [834 836]
[864 868] is a split TAD from [864 873]
[869 897] is a merged TAD from [874 897]
[898 899] is a split TAD from [898 922]
[900 923] is a split TAD from [898 922]
[924 972] is a matched TAD with [923 973]
[973 982] is a matched TAD with [974 982]
[983 996] is a matched TAD with [983 997]
[ 997 1011] is a merged TAD from [ 998 1009]
[1012 1337] is a shifted TAD of [1010 1333]
[1338 1354] is a split TAD from [1334 1354]
[1355 1362] is a merged TAD from [1355 1357]
[1363 1363] is a split TAD from [1358 1362]
[1364 1370] is a merged TAD from [1363 1363]
[1371 1372] is a split TAD from [1369 1372]
[1373 1374] is a split TAD from [1373 1377]
[1375 1379] is a merged TAD from [1378 1379]
[1380 1395] is a matched TAD with [1380 1395]
[1396 1413] is a matched TAD with [1396 1414]
[1414 1415] is a split TAD from [1396 1414]
[1416 1489] is a merged TAD from [1415 1423]
[1490 1492] is a split TAD from [1490 1494]
[1493 1494] is a split TAD from [1490 1494]
[1495 1496] is a matched TAD with [1495 1496]
[1497 1498] is a matched TAD with [1497 1498]
[  1 819] is a merged TAD from [  1 272]
[ 820 1498] is a merged TAD from [821 833]
#match = 11 #merge = 14 #split = 15 #shift = 1
```

B

RNAs related to common TLDs on chromosome 6 in H3K27me3_RADIP dataset (41 RNAs)

Zfp777
Bola3
Dera
Tgfa
Mturn
Uba3
Pde3a
Sinhcaf
Bicd1
Hoxa11
Gm36640
Mthfd2
Slc4a5
Arl6ip5
Rbsn
Plekha8
Dennd5b
Zfp746
Gm28308
Pdia4
Frmd4b
Adcyap1r1
Rerg
Gcfc2
Tet3
Hoxaas3
Mob1a
Snrpg
Emp1
Resf1
Txnrd3
Gm765
Grin2b
Hoxa11os
Gm20696
Ptpro
Slc15a5
Ipo8
Cntnap2
Creb5
Hoxa10

RNAs related to changed TLDs on chromosome 6 in H3K27me3_RADIP dataset (28 RNAs)

Pde6h
Foxp1
Vps50
Uba3
Pde3a
Wipf3
Plekha8
Eogt
Gmcl1
Dennd5b
Jazf1
Sspo
Gm43948
Anxa4
Adcyap1r1
Rerg
Prickle2
Zfp282
Krba1
Aak1
Gm20696
Asns
Pdzrn3
Scrn1
Tmf1
Amn1
Eps8
Gm43950

Figure S6. TLDs (topologically associating domain-like domains) for comparison of Input samples and H3K27me3-RADIP samples.

(**A**) TLDs on chromosome 6 and the details of changes in TLDs on chromosome 6 for both types of samples are shown. (**B**) We identified and collected the anchor information for common TLDs and changed TLDs on chromosome (chr.) 6. We then determined which RNA species were related to these common TLDs (left) and changed TLDs (right) on chr. 6 in H3K27me3-RADIP samples as an example. Next, we followed this strategy for all chromosomes to determine the "Changed TLD" RNAs on a genome-wide level.

Figure S7.

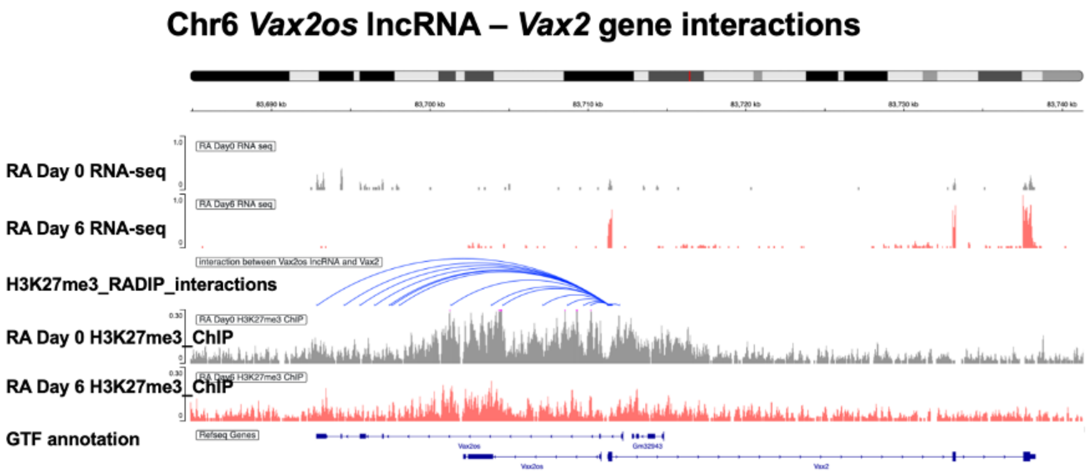

Figure S7. An additional example of RNA-mediated lineage-specific transcriptional silencing.

Visualization of interactions between *Vax2os* lncRNA and the *Vax2* gene. the *Vax2os* lncRNA guides PRC2 to the TSS of the *Vax2* gene, facilitating the deposition of H3K27me3 and thereby silencing the *Vax2* gene in mESCs. As differentiation progresses, the expression of *Vax2os* lncRNA ceases, leading to diminished guidance, reduced PRC2 localization, and a consequent decrease in H3K27me3 intensity at the TSS of *Vax2* gene. These changes result in a significant up-regulation of *Vax2* gene expression, which is critical for forebrain development.

Figure S8.

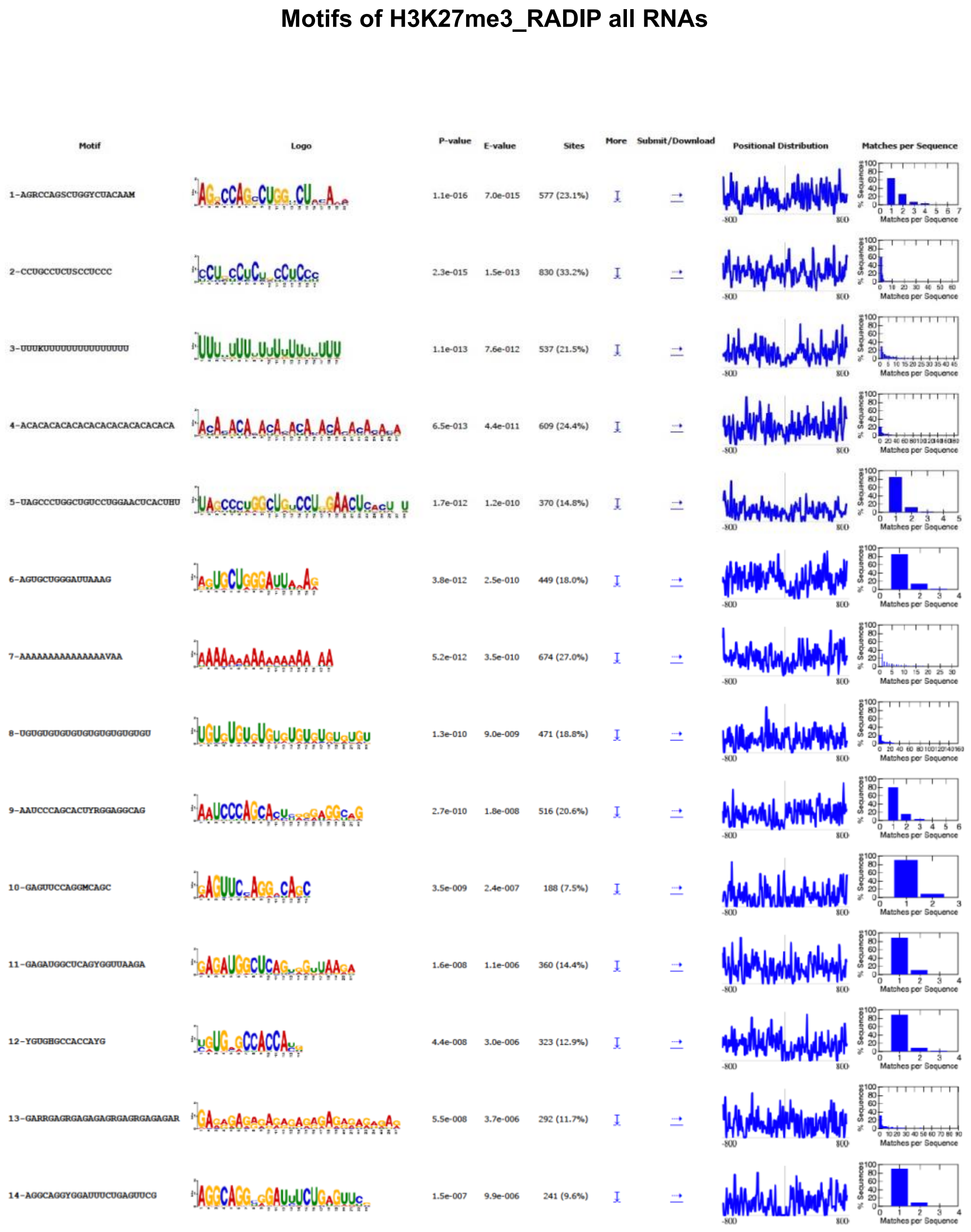

Figure S8. Full list of enriched motifs from all RNA tags of H3K27me3-RADIP samples (page 1).

Figure S9.

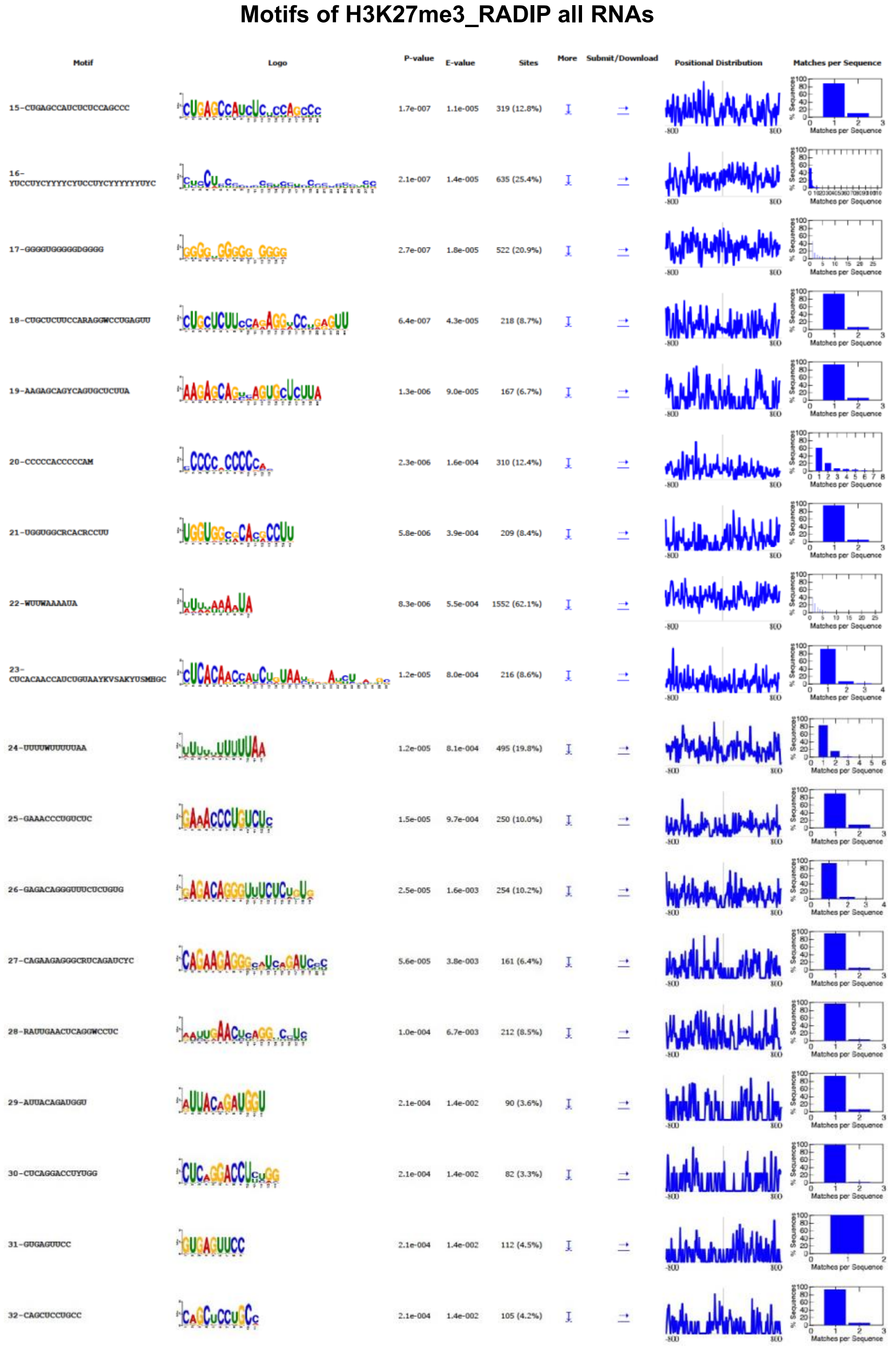

Motifs of H3K27me3_RADIP all RNAs

Motif	P-value	E-value	Sites
15-CUGAGCCAUCUCUCCAGCCC	1.7e-007	1.1e-005	319 (12.8%)
16-YUCCUYCYYYYCYUCCUYCYYYYYYUYC	2.1e-007	1.4e-005	635 (25.4%)
17-GGGGUGGGGGDGGGG	2.7e-007	1.8e-005	522 (20.9%)
18-CUGCUCUUCCARAGGWCCUGAGUU	6.4e-007	4.3e-005	218 (8.7%)
19-AAGAGCAGYCAGUGCUCUUA	1.3e-006	9.0e-005	167 (6.7%)
20-CCCCCACCCCCAM	2.3e-006	1.6e-004	310 (12.4%)
21-UGGUGGCRCACRCCUU	5.8e-006	3.9e-004	209 (8.4%)
22-WUUWAAAAUA	8.3e-006	5.5e-004	1552 (62.1%)
23-CUCACAACCAUCUGUAAYKVSAKYUSMHGC	1.2e-005	8.0e-004	216 (8.6%)
24-UUUUWUUUUUAA	1.2e-005	8.1e-004	495 (19.8%)
25-GAAACCCUGUCUC	1.5e-005	9.7e-004	250 (10.0%)
26-GAGACAGGGUUUCUCUGUG	2.5e-005	1.6e-003	254 (10.2%)
27-CAGAAGAGGGCRUCAGAUCYC	5.6e-005	3.8e-003	161 (6.4%)
28-RAUUGAACUCAGGWCCUC	1.0e-004	6.7e-003	212 (8.5%)
29-AUUACAGAUGGU	2.1e-004	1.4e-002	90 (3.6%)
30-CUCAGGACCUYUGG	2.1e-004	1.4e-002	82 (3.3%)
31-GUGAGUUCC	2.1e-004	1.4e-002	112 (4.5%)
32-CAGCUCCUGCC	2.1e-004	1.4e-002	105 (4.2%)

Figure S9. Full list of enriched motifs from all RNA tags of H3K27me3-RADIP samples (page 2).

Figure S10.

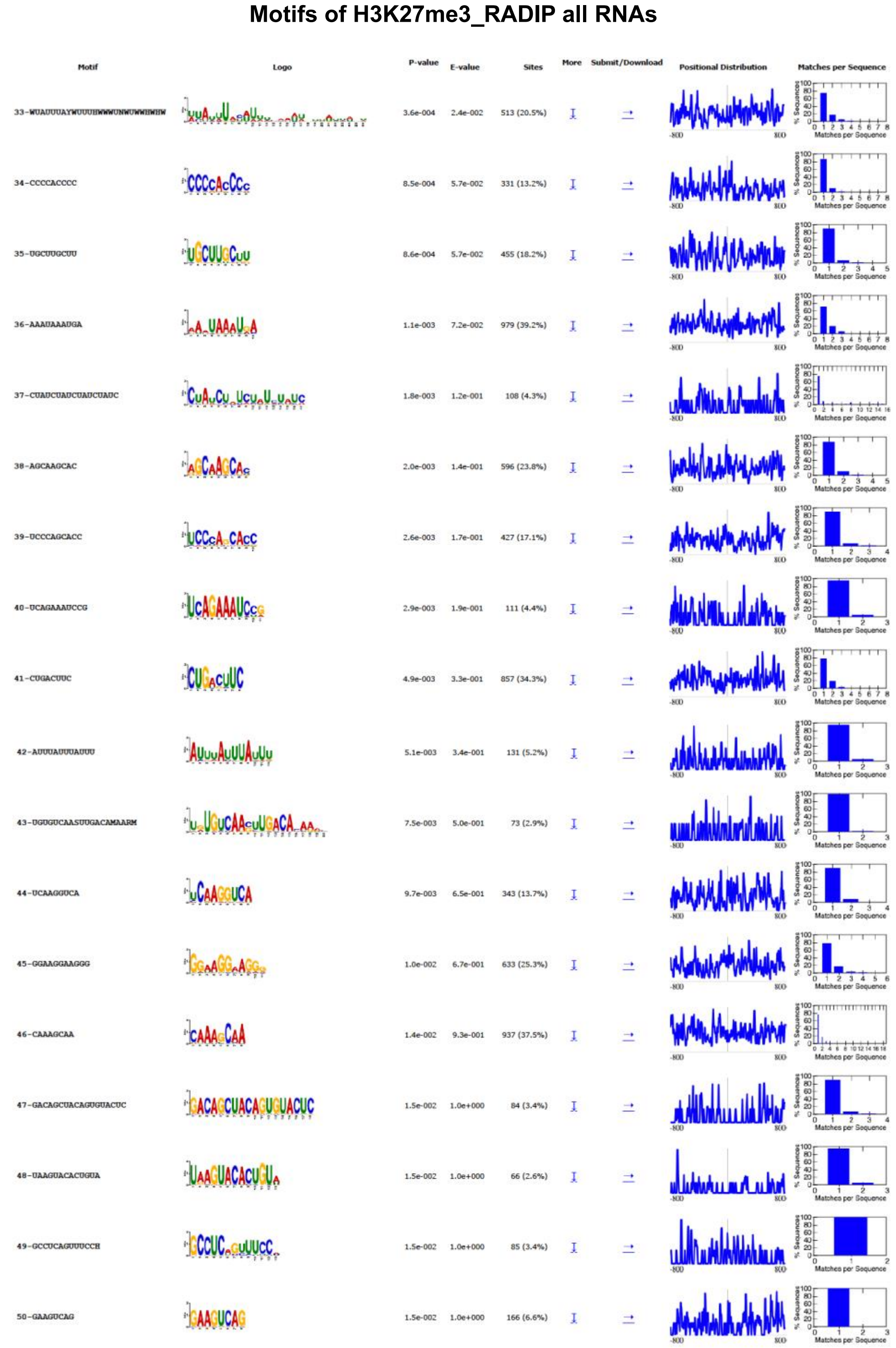

Figure S10. Full list of enriched motifs from all RNA tags of H3K27me3-RADIP samples (page 3).

Figure S11.

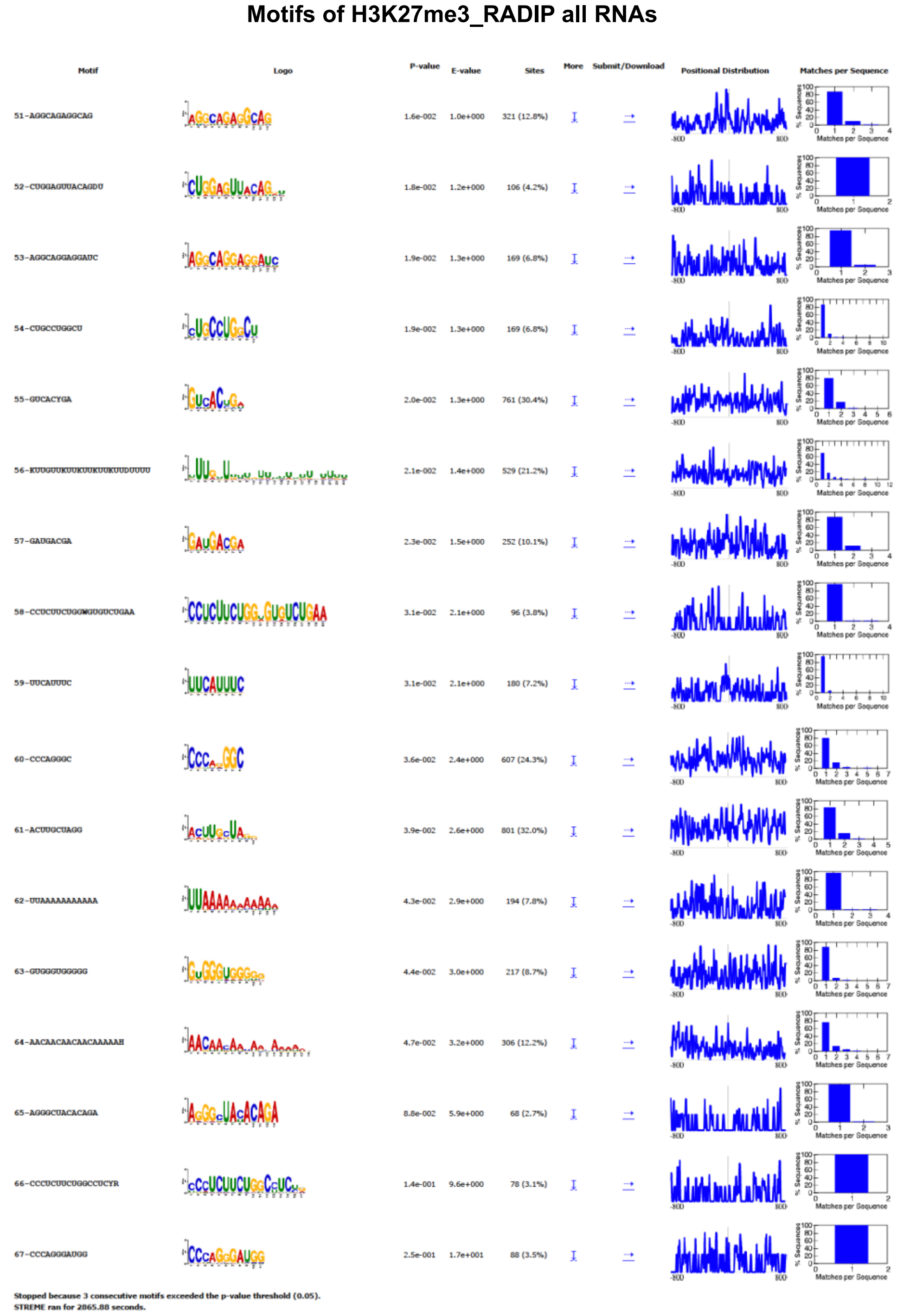

Figure S11. Full list of enriched motifs from all RNA tags of H3K27me3-RADIP samples (page 4).

Figure S12.

Motifs of H3K27me3_RADIP unique RNAs

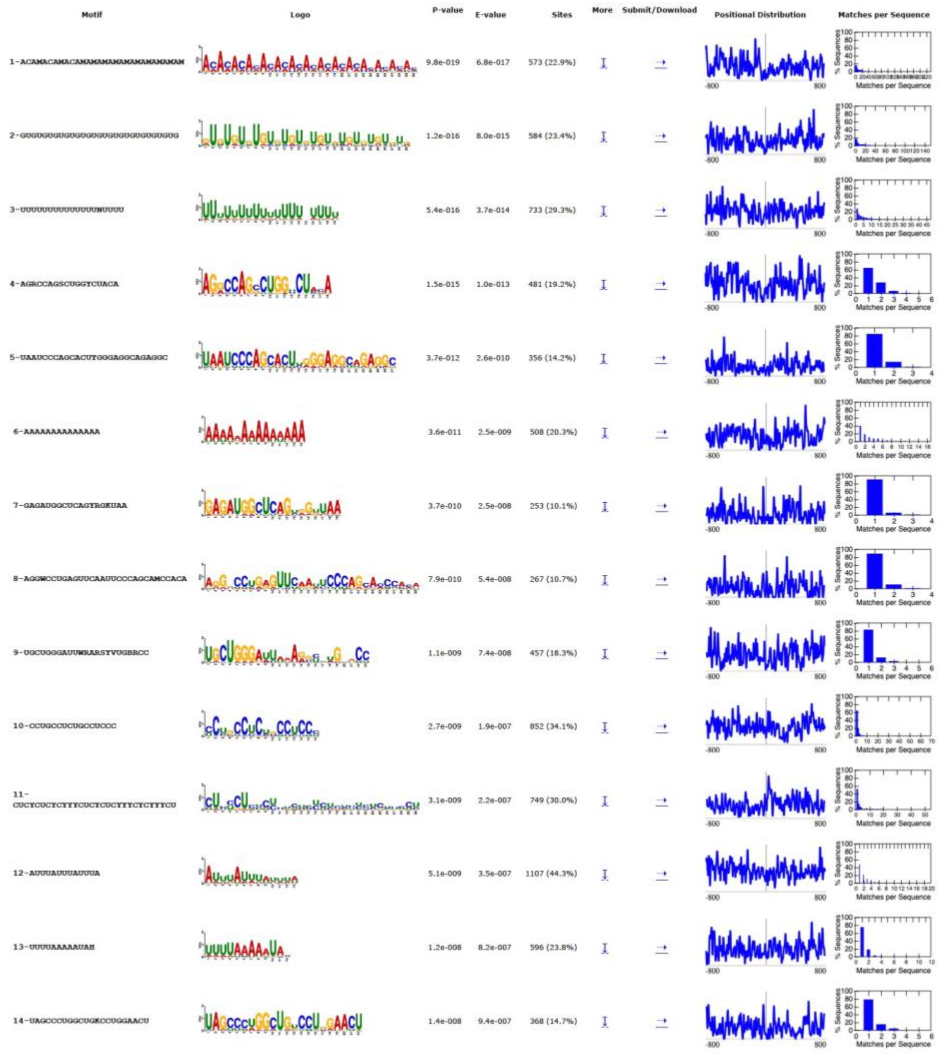

Figure S12. Full list of enriched motifs from RNA tags unique to H3K27me3-RADIP samples (page 1).

Figure S13.

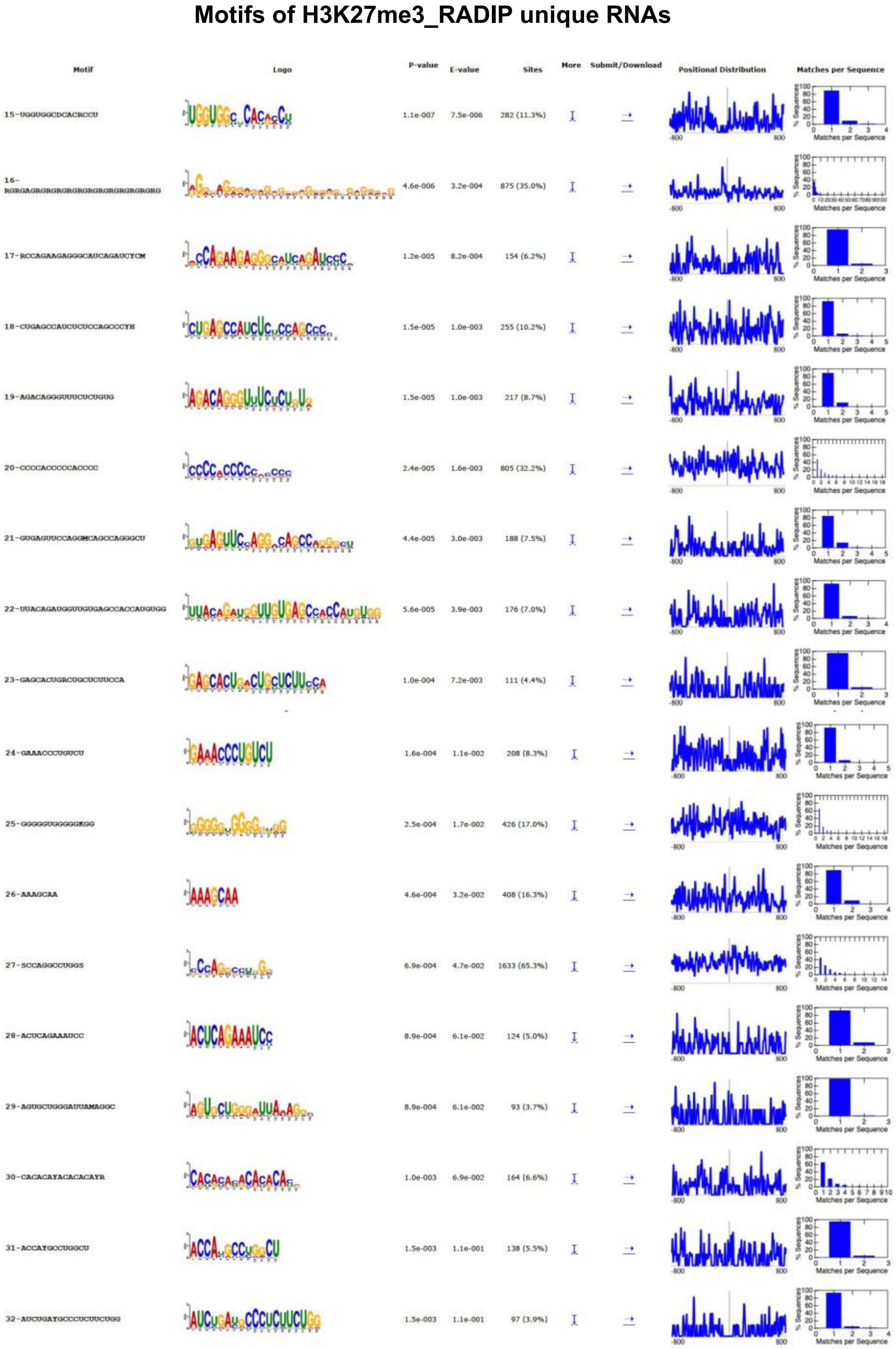

Figure S13. Full list of enriched motifs from RNA tags unique to H3K27me3-RADIP samples (page 2).

Figure S14.

Motifs of H3K27me3_RADIP unique RNAs

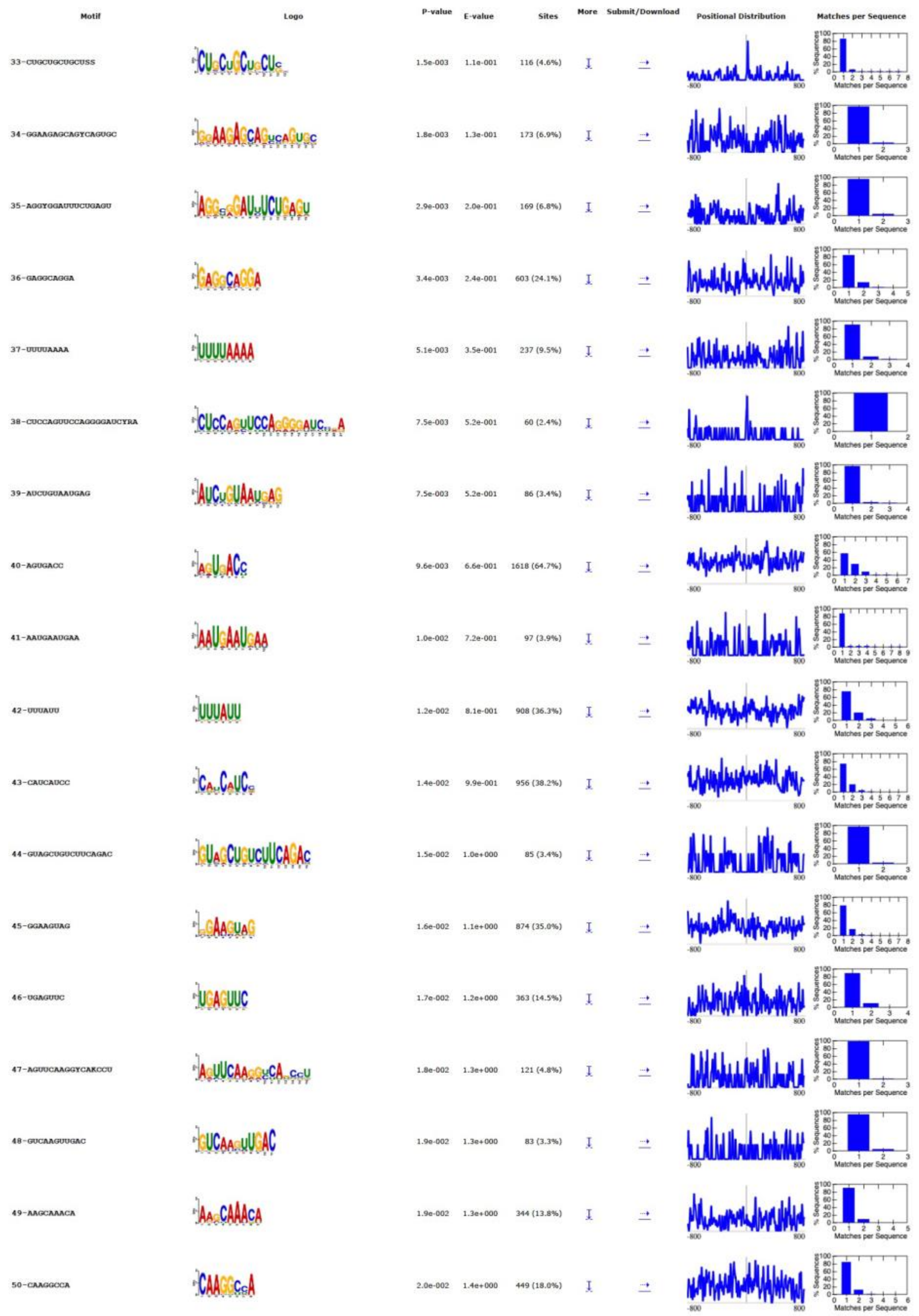

Motif	P-value	E-value	Sites
33-CUGCUGCUGCUSS	1.5e-003	1.1e-001	116 (4.6%)
34-GGAAGAGCAGYCAGUGC	1.8e-003	1.3e-001	173 (6.9%)
35-AGGYGGAUUUCUGAGU	2.9e-003	2.0e-001	169 (6.8%)
36-GAGGCAGGA	3.4e-003	2.4e-001	603 (24.1%)
37-UUUUAAAA	5.1e-003	3.5e-001	237 (9.5%)
38-CUCCAGUUCCAGGGGAUCYRA	7.5e-003	5.2e-001	60 (2.4%)
39-AUCUGUAAUGAG	7.5e-003	5.2e-001	86 (3.4%)
40-AGUGACC	9.6e-003	6.6e-001	1618 (64.7%)
41-AAUGAAUGAA	1.0e-002	7.2e-001	97 (3.9%)
42-UUUAUU	1.2e-002	8.1e-001	908 (36.3%)
43-CAUCAUCC	1.4e-002	9.9e-001	956 (38.2%)
44-GUAGCUGUCUUCAGAC	1.5e-002	1.0e+000	85 (3.4%)
45-GGAAGUAG	1.6e-002	1.1e+000	874 (35.0%)
46-UGAGUUC	1.7e-002	1.2e+000	363 (14.5%)
47-AGUUCAAGGYCAKCCU	1.8e-002	1.3e+000	121 (4.8%)
48-GUCAAGUUGAC	1.9e-002	1.3e+000	83 (3.3%)
49-AAGCAAACA	1.9e-002	1.3e+000	344 (13.8%)
50-CAAGGCCA	2.0e-002	1.4e+000	449 (18.0%)

Figure S14. Full list of enriched motifs from RNA tags unique to H3K27me3-RADIP samples (page 3).

Figure S15.

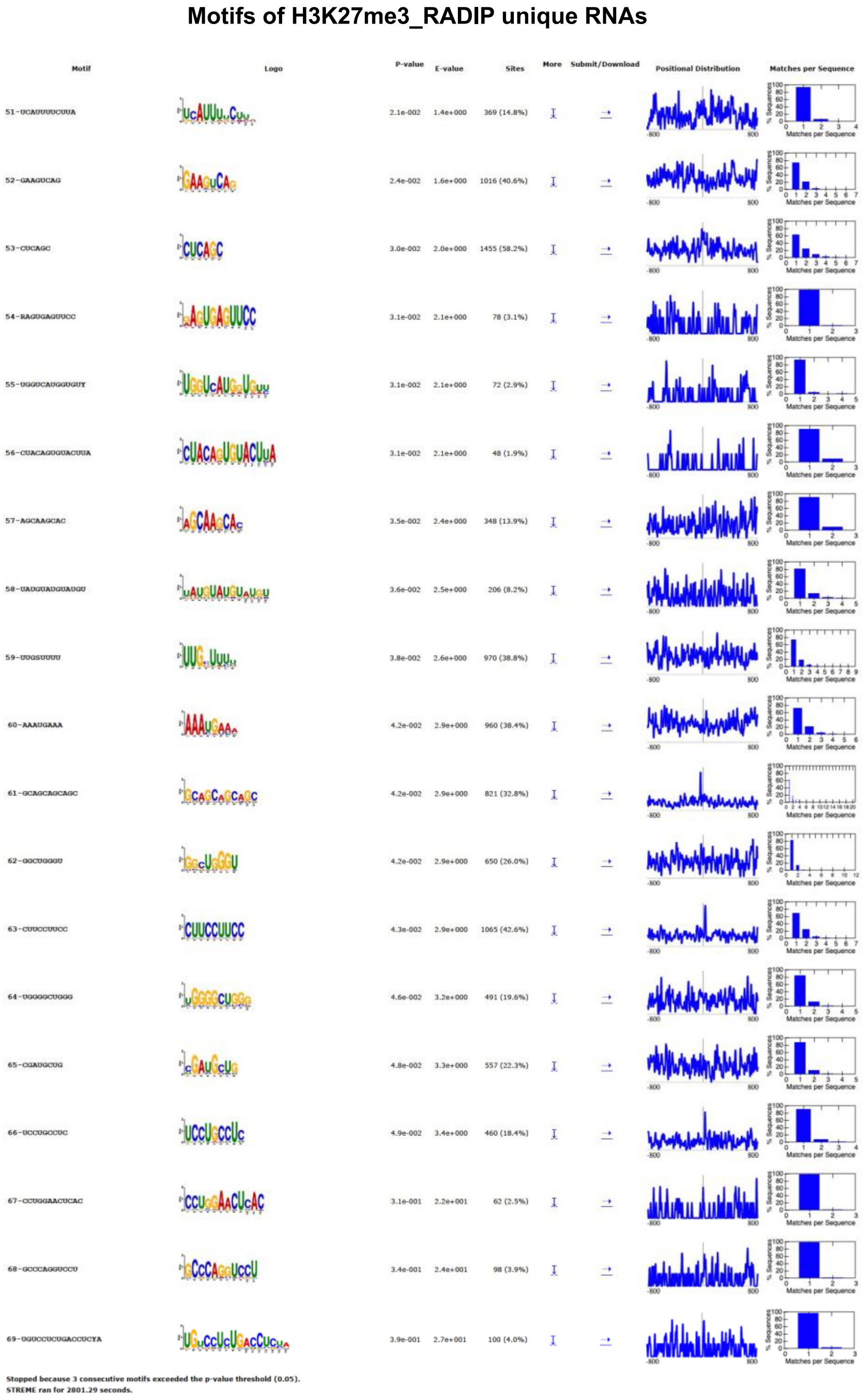

Figure S15. Full list of enriched motifs from RNA tags unique to H3K27me3-RADIP samples (page 4).

Figure S16.

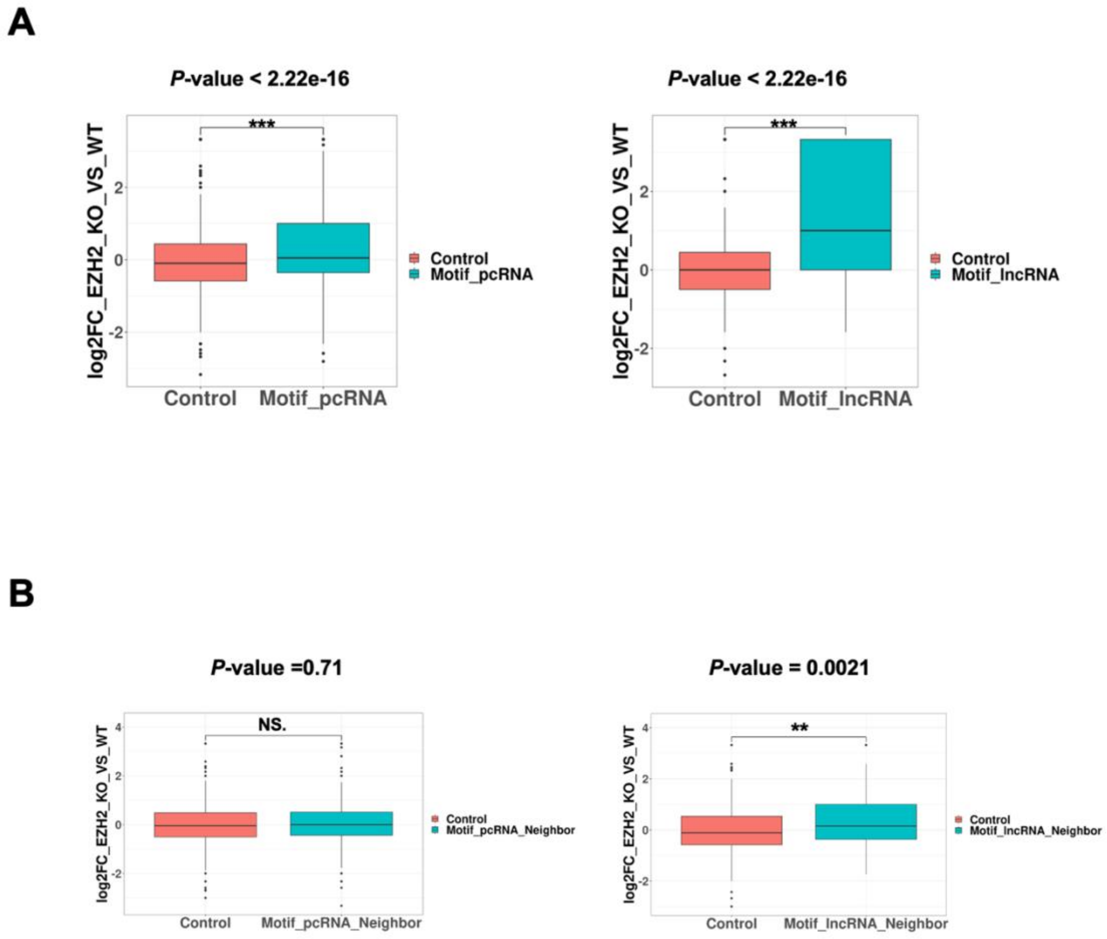

Figure S16. Expression-level changes of motif-containing RNAs and neighboring genes after EZH2 knockout.

(**A**) The expression levels of RNAs containing GCA and CUG repeat motifs were significantly elevated compared to control RNAs (randomly selected and numerically equivalent) after EZH2 knockout. This result suggests that these motif-containing RNAs contribute to the recruitment of PRC2 to their genomic regions, leading to H3K27me3 histone mark deposition and gene repression. (**B**) The expression levels of neighboring genes around source lncRNA genes were significantly increased, while the expression levels of neighboring genes around source pcRNA genes remained unchanged. This suggests that GCA-repeat- and CUG-repeat-motif-containing lncRNAs facilitate the spreading of PRC2 and H3K27me3 histone marks across surrounding regions, whereas motif-containing pcRNAs confine PRC2 recruitment to their gene loci.

Figure S17.

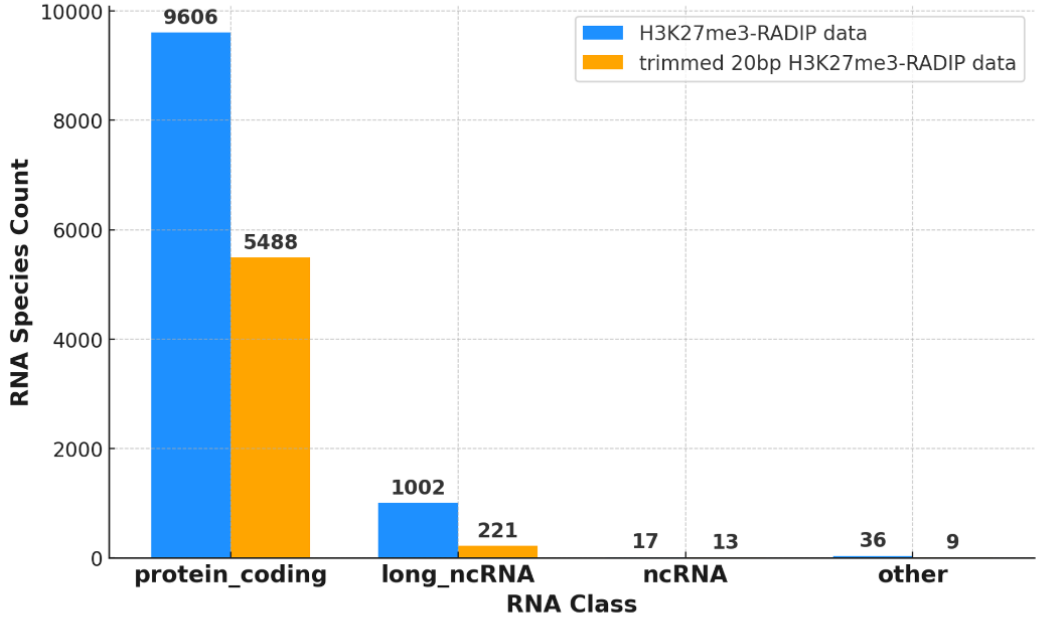

Figure S17. Comparison of RNA detection between H3K27me3-RADIP and trimmed 20bp H3K27me3-RADIP.

The H3K27me3-RADIP captured approximately 1.8 times more pcRNAs and about 4.5 times more lncRNAs compared to the trimmed 20bp version. This result underscores the significant impact of improved genome coverage and a higher proportion of uniquely mapped reads on the enhanced detection of various RNA types, particularly lncRNAs, which are crucial for investigating PRC2-associated lncRNA-DNA interactions and their role in chromatin regulation.
