# Supplementary material for: RADIP technology comprehensively identifies H3K27me3-associated RNA–chromatin interactions
Source: Nucleic Acids Res. 2024 Nov 18;52(22):e104. doi: 10.1093/nar/gkae1054 (PMC11662664; doi:10.1093/nar/gkae1054)
Supplement: gkae1054_Supplemental_Files [file gkae1054_supplemental_files.zip › Resubmit Shu RADIP_NAR_format_Supplementary Methods_20241011.pdf]

SUPPLEMENTARY METHODS

GSK126 treatment and cell fractionation. Cells were treated with 0.1% DMSO (control) or 5 µM GSK126 (Merck) for 48 h. To extract the cytoplasmic, nuclear, and chromatin fractions, we followed a protocol described previously[1].

RNA extraction, cDNA synthesis, and quantitative RT-PCR. RNA was isolated using RNeasy Plus Mini Kit (Qiagen). An on-column DNase I (New England Biolabs) digestion was performed in accordance with the manufacturer's instructions. For chromatin RNA, we added 250 µL of precooled PBS to the chromatin pellet and resuspended the pellet by pipetting. We then added 750 µL of TRIzol™ LS Reagent (Invirtogen) with 10 µL of 0.5 M EDTA and incubated the mixture at 65 °C for about 20 min, with vortexing. Next, we added 200 µL of chloroform, vortexed the mixture briefly, and incubated it at room temperature for 5 min. This was followed by centrifugation for 15 min at 16,000*g* and 4 °C.

We transferred the upper aqueous layer to a new tube and added 3.5 sample volumes of Buffer RLT (Qiagen) We then followed the cleanup protocol of the RNeasy Kit. An equivalent volume of total RNA for each fraction was used for cDNA synthesis by using a SuperScript IV kit (Thermo Fisher Scientific) with Random 6-mer primers. For qRT-PCR, a PowerTrack SYBR Kit (Thermo Fisher Scientific) was used. The amount of RNA relative to that of HPRT (hypoxanthine phosphoribosyltransferase) in the cytoplasm fraction was calculated for each fraction, and then the percentage of RNA in the chromatin fraction was calculated.

The qRT-PCR primer sequences were as follows:
Airn_intron_Fw: GGGTCGAATCAGGTCACACA
Airn_intron_Rv: TCTCTGCTCTTGTGGGCAAA
Airn_ex1-2_Fw: CAGACATCCTGGGGAACTGA
Airn_ex1-2_Rv: CTGGCAACCTGCCCTATTTC
Nanog_intron_Fw: CCGTTTTCTCCAGCAGAAGC
Nanog_intron_Rv: TGTTTGGGGTTTGGAAGGAG
Nanog_exon_Fw: TTCTGGGAACGCCTCATCAATGCCTGC
Nanog_exon_Rv: TCCTCAGGGCCCTTGTCAGCCTCAG
Lncppara_intron_ Fw: GGAAAGGCAGCAGAAAAGGA
Lncppara_intron_ Rv: GAAGGAACGTGTGACGGACA
Lncppara_ex5-6_ Fw: TTGACAACTTCCCTGGGTGA
Lncppara_ex5-6_ Rv: AAGGCAGCCGGTTGTACTGT
2500002B13_intron_Fw: CAGAGGGGCCTCATATCTCTG

2500002B13_intron_Rv: AAGGACCATAGCCCCCAAGT

2500002B13_exon_Fw: TTCAGACCGCCAGCTGACTC

2500002B13_exon_Rv: CACTTTCATCTGAAGGTTGCTATG

Hprt_exon_Fw: CAGGCCAGACTTTGTTGGAT

Hprt_exon_Rv: TTGCGCTCATCTTAGGCTT

Details of modified CHiCANE method used to select significant pairs. A modified negative binomial distribution approach, as implemented with the CHiCANE package in R, was used to calculate the adjusted *P*-value (*q* value) for each interaction pair. Then, only statistically significant interaction reads (q value ≤ 0.1) were retained for subsequent analysis.

The purpose-defined CHiCANE-based method merely considers the intractability of the RNA tags. We used *trans* RNA–DNA interactions to produce the background.

Let Y_{ij} denote the number of reads linking RNA tag $_i$ and target DNA tag $_j$, and let t_i denote the number of reads linking the RNA tag $_i$ with all target DNA tags in *trans*. μ is a mean parameter, and θ is a dispersion parameter calculated from the count of all interaction pairs. After β_0, β_1, and the overdispersion term are fitted by the maximum likelihood method over the count of all interaction pairs, the MLEs (maximum likelihood estimates) of the coefficients β_0 and β_1 are used to provide an estimate of the new expected mean parameter μ_{ij} for each interaction pair, where:

$$\mu_{ij} = \beta_0 + \beta_1 \log(t_i + 1)$$

The MLE of the dispersion parameter θ is used as the dispersion parameter in the negative binomial model without zero observations (zero-count bins), where:

$$Y \sim \mathrm{NB}(\mu, \theta)$$

Therefore, Y_{ij} is assumed to follow the fitted NB (negative binomial) regression with the adjusted mean parameter μ_{ij} and the adjusted dispersion parameter θ_{ij} for each interaction pair:

$$\mathrm{Var}(Y_{ij}) \sim \mu_{ij} + (\mu_{ij}^2 / \theta_{ij})$$

Next, a P value for each interaction pair can be estimated for the observed counts y_{ij} exceeding what is expected from the fitted NB model (background/noise), as:

$$P = P(Y_{ij} \geq y_{ij})$$

Finally, a multiple testing correction (by the Benjamini and Hochberg method) was applied to convert the P value into a q value (adjusted P value or false discovery rate, or both) and identify significant interaction pairs that interacted more frequently than expected by chance, with a cutoff q value of no more than 0.1. Only the significant datasets were used for subsequent analysis.

1. Sharma, H. et al. Decryption of sequence, structure, and functional features of SINE repeat elements in SINEUP non-coding RNA-mediated post-transcriptional gene regulation. *Nat Commun* **15**, 1400 (2024).
