# Supplementary material for: RADIP technology comprehensively identifies H3K27me3-associated RNA–chromatin interactions
Source: Nucleic Acids Res. 2024 Nov 18;52(22):e104. doi: 10.1093/nar/gkae1054 (PMC11662664; doi:10.1093/nar/gkae1054)
Supplement: gkae1054_Supplemental_Files [file gkae1054_supplemental_files.zip › Resubmit Shu RADIP_NAR_format_Supplementary Protocol_20241011.pdf]

RADIP protocol

CELL CROSSLINKING

- Trypsinize cells. After neutralizing the trypsin with media, transfer into falcon tube
- Spin 500 xg for 5 minutes
- Discard supernatant
- Resuspend cell pellet in PBS or HBSS
- Spin 500 xg for 5 minutes
- Discard supernatant
- Resuspend cell pellet in PBS (1 ml every 2 million cells)
- Add equal amount of 2 % formaldehyde (stock: 16% Formaldehyde (w/v), Methanol-free) diluted with PBS. Note: final FA concentration is 1%
- Incubate cells at room temperature (RT) for 10 minutes with slow rotation
- Add 1M glycine solution (final concentration 125 mM)
- Incubate cells at RT for 5 minutes with slow rotation
- Centrifuge the cells at no more than 150 xg for 5 minutes at 4 °C, remove the supernatant and wash the pellet with ice-cold PBS.
- Centrifuge the cells at no more than 150 xg for 5 minutes at 4 °C, remove the supernatant and snap frozen in liquid nitrogen
- Store at -80˚C

Day 1

CELL LYSIS

Lyse plasma membrane in hypotonic buffer, isolate nuclei, permeabilize nuclear membrane

- Prepare 4 ml lysis buffer in 50 ml falcon (400 µl every 2 million cells)
- Get 800 µl lysis buffer and add to frozen pellet, gently resuspend
- Transfer the resuspended pellet back to the original 50 ml falcon
- Gently resuspend
- Keep on ice 10 minutes
- Dispense 400 µl in 10 1.5 ml tubes previously put on ice.
- Spin 500 xg 4 minutes at 4°C - swinging rotor
- Discard supernatant
- Resuspend in DNase I digestion buffer I 100 µl (gently pipetting)
- Shake 850 rpm 30 minutes at 37°C on Thermomixer
- Add 100 µl DNase I digestion buffer II
- Shake 850 rpm 10 minutes at 37°C on Thermomixer

DNase I DIGESTION

DNase I digestion timing for this batch of mESCs (PGK12.1)

Add 1.5 µl DNase I (1U/ µl) for 4 minutes*.

**The optimal DNase I digestion time varies depending on the cell pellet batch and cell type. The average fragment size should be approximately 1 kilobase (1 kb).*

- Stop the digestion with 40 µl DNase I stop buffer at the indicated time points.
- Pellet nuclei 2,500 xg 4 minutes at RT - swinging rotor

PELLET WASH

- Remove supernatant
- Resuspend pellet with the mixed buffer of the following reaction (200µl/tube)

10x T4 DNA ligation buffer	20 µl
10mM dNTPs	5 µl
H_2O	175 µl

- Spin 2,500 xg 4 minutes at RT - swinging rotor
- Remove supernatant

DNA END REPAIR

- Prepare the following mixture (per tube)

10x T4 DNA ligation buffer	20 µl
10mM dNTPs	5 µl
T4 DNA Polymerase (5U/µl) (Thermo)	3 µl
Klenow Fragment (10U/µl)	3 µl
H_2O	169 µl

- Resuspend the nuclear pellet in 200 µl
- Shake 850 rpm 1h at 25°C (RT) on Thermomixer
- Stop reaction by adding 5 µl 10% SDS

PELLET WASH

- Spin 2,500 xg 4 minutes at RT - swinging rotor
- Remove supernatant
- Resuspend pellet with the mixed buffer of the following reaction (185 µl/tube)

10x NEB buffer 2	20 µl
10mM dATP	10 µl
10% Triton X-100	20 µl
H_2O	135 µl

- Spin 2,500 xg 4 minutes at RT - swinging rotor
- Remove supernatant

dA-TAILING

- Resuspend the pellet in 185 µl of the dA tailing buffer showed above
- Add 15 µl of Klenow Fragment (exo-)
- Shake 850 rpm 1h at 37°C on Thermomixer

RNase H TREATMENT

- Add 5 µl RNase H (5,000U/ml, final concentration 0.122 U/µl)
- Shake 850 rpm 40 minutes at 37°C on Thermomixer

PELLET WASH

- Spin 2,500 xg 4 minutes at RT - swinging rotor
- Remove supernatant
- Resuspend pellet with the mixed buffer of the following reaction (200 µl/tube)

10x T4 RNA ligation buffer	20 µl
H_2O	180 µl

- Spin 2,500 xg 4 minutes at RT - swinging rotor
- Remove supernatant

BRIDGE ADAPTOR AND RNA LIGATION

- Prepare the RNA ligation mixture (per tube)

10x T4 RNA ligation buffer	3 µl
Pre-adenylated adaptor	1 µl
RNase OUT	1 µl
T4 RNA ligase 2, truncated KQ	2 µl
H_2O	23 µl

- Resuspend pellet in 30 µl
- Shake 850 rpm O/N at 20°C on Thermomixer

Day 2

PELLET WASH

- Stop reaction by adding 170 µl Triton Solution

10% Triton X-100	20 µl
H_2O	150 µl

- Spin 2,500 xg 4 minutes at RT - swinging rotor
- Remove supernatant
- Resuspend pellet with the mixed buffer of the following reaction (200 µl/tube)

10x T4 DNA ligation buffer	20 µl
H_2O	180 µl

- Spin 2,500 xg 4 minutes at RT - swinging rotor
- Remove supernatant

BRIDGE ADAPTOR AND DNA LIGATION

- Resuspend pellet in 495 µl DNA ligation reaction buffer showed above
- Add 5 µl T4 DNA ligase (NEB, 400U/µl), total reaction volume 500 µl
- Shake 850 rpm 4h at 25°C on Thermomixer

DYNABEADS IgG EQUILIBRATION & ANTIBODY COUPLING

The volumes reported here are intended for 5 tubes

- Collect 30 µl of Dynabeads M-280 mouse or rabbit IgG
- Wash beads with 500 µl of ChIP dilution buffer
- Resuspend beads in 15 µl ChIP dilution buffer
- Add 3-5 µg of antibody
- Vortex periodically to resuspend beads and stand on ice at least 1h
- Wash beads twice with 500 µl ChIP dilution buffer when ready to proceed with the following O/N incubation

STOP DNA LIGATION & BRIEF SONICATION

A brief sonication is required only to break the nuclear membrane

- Stop the ligation reaction by spinning 2,500 xg 4 minutes at RT - swinging rotor
- Remove supernatant
- Resuspend pellet in 20 µl SDS lysis buffer

- Pool the 5 tubes in 1 tube, total volume 100 µl.
- Keep on ice for 10 minutes
- Add 400µl ChIP dilution buffer, total volume 500 µl
- Split into 2 Picoruptor tubes (250 µl each)
- Sonicate with Picoruptor (Diagenode) 1 cycle of 5 seconds ON + 30 seconds OFF at 4°C
- Transfer into 1 1.5 ml tube
- Centrifuge max speed 5 minutes at 4°C
- Collect supernatant of 500 µl
- Add 600 µl ChIP dilution buffer, total volume 1.1ml
- Move 100 µl to a new tube to be used for Input library preparation
    - Add 25 µl Proteinase K buffer
    - Add 11 µl Proteinase K
    - De-crosslink by shaking 850rpm O/N at 65°C on a Thermomixer
- Transfer left 1ml to the previously prepared and washed beads
- Incubate by rotating O/N at 4°C

Day 3

PURIFICATION OF INPUT BY PCR PURIFICATION KIT

- Elute in 30 µl, 5 µl used for Qubit & Bioanalyzer later, 25µl kept being processed and sequenced
- Pool the 2 technical replicates of Input together, total volume 50 µl
- Freeze at -80°C

BEADS WASHING AND DE-CROSSLINKING

- Wash beads 3 times with 500 µl RIPA 500mM buffer
- Resuspend beads in 200 µl water
- Add 50 µl Proteinase K buffer and 22µl Proteinase K
- Shake 850rpm 5 h at 65°C on Thermomixer

PURIFICATION OF RADIP BY PCR PURIFICATION KIT

- Put tubes on the magnet, collect supernatant
- Purify the supernatant by PCR purification Kit, elution volume 30 µl
- Pool the 2 technical replicates of RADIP together, total volume 60 µl
- Freeze at -80°C

Day 4

The steps for preparing both Input and RADIP libraries are now the same. Follow these steps to prepare either an Input or RADIP library:

CONCENTRATION

- Speedvac to 12 µl at 37°C

REVERSE TRANSCRIPTION

- Add 1µl 10mM dNTPs
- Incubate 5 minutes at 65°C
- Promptly put on ice
- Meanwhile prepare the following reverse transcription mixture (SSIV Thermo):

5x First strand buffer	4 µl
0.1M DTT	1 µl
RNaseOUT	1 µl

SuperScript IV	1 µl

- Run reverse transcription program:

56°C	10 min
80°C	10 min
4°C	hold

SECOND STRAND SYNTHESIS

- Prepare the following reaction mix

5x Second Strand buffer	30 µl
10mM dNTPs	3 µl
RNaseH (2U/µl) Thermo	3 µl
E coli DNA polymerase I (10U/µl) NEB	4 µl
E coli DNA ligase (10U/µl)	1 µl
H_2O	89 µl

- Incubate 16°C 2h in thermal cycler
- Stop reaction by adding 10µl 0.5M EDTA

PURIFICATION BY PCR PURIFICATION KIT

- Elute in 30 µl H_2O

QUBIT HIGH SENSITIVITY KIT

Determine the concentration to calculate the amount of EcoP15I required for the following EcoP15I digestion step (Use 10U Ecop15I for 1.5 µg DNA).

ECOP15I DIGESTION

- Prepare the following digestion mixture

NEB 3.1 buffer	5 µl
10x ATP for EcoP15I	5 µl
10mM Sinefungin	0.5 µl

- Calculate the volume of **EcoP15I (10U/µl)** and water to add, final volume: 50 µl
- Incubate O/N at 37°C (no shaking)

Day 5

PURIFICATION BY NUCLEOTIDE REMOVAL KIT (QIAGEN)

- Elute in 50 µl EB

END REPAIR AND Y-SHAPED ADAPTOR LIGATION

- Add 7 µl NEBNext Ultra II End Prep Reaction Buffer and 3µl of NEBNext Ultra II End Prep Enzyme mix
- Thermal cycle program

20°C	30 mins
65°C	30 mins
4°C	hold

- Prepare the following reaction mixture

NEBNext Ultra II Ligase Master mix	30 µl
10µM Y-shaped adaptors	2 µl
NEBNext Ligation Enhancer	1 µl

- Final volume: 93 µl
- Incubate 15 minutes at 20°C

STREPTAVIDIN BEADS CAPTURE

- Equilibration of MyOne C1 Streptavidin Beads (Dynabeads Thermo)
  - Wash 20 µl beads once with 200µl 1x WB buffer
  - Wash beads one more time with 200 µl 2x WB buffer
  - Resuspend in 93 µl 2x WB buffer
- Add 93µl of bead suspension to 93µl of sample at the end of ligation
- Rotate 20 minutes at RT
- Wash 3 times with 200 µl 1x WB buffer
- Wash once with EB
- Resuspend in 30 µl EB

PCR CYCLE TEST

Three amplification cycle conditions (8, 11, and 14 cycles) are tested using the Phusion High Fidelity PCR Kit (Thermo Fisher Scientific) with 4 µl of the isolated sample. After 8, 11, and 14 cycles respectively, 10 µl aliquots were collected and run on a pre-cast 6% polyacrylamide gel (Invitrogen) at 145 V for 60 minutes. The lowest PCR cycle at which the 220bp to 225bp band, representing the RNA-DNA ligated complexes, could be visualized was chosen for the final library amplification. The goal is to obtain enough material for sequencing while minimizing PCR duplicates.

- Freeze at -20°C

Day 6

LIBRARY PCR AMPLIFICATION

After selecting the most appropriate cycle number, amplify the rest of the sample. Following amplification, the six reactions were pooled and purified using the PCR purification Kit with an elution volume of 30 µl. The 30 µl of the library was then run on a pre-cast 6% polyacrylamide gel (Invitrogen) at 145 V for 60 minutes. The 220bp to 225bp band was excised and purified.

- Freeze at -20°C

Day 7

LIBRARY SEQUENCING

Library was quantified by quantitative PCR with the Library Quantification Kit for Illumina sequencing platforms (KAPA Biosystems) on a StepOne Real-Time PCR System (Applied Biosystems). Sequencing was performed using a Single-End 150bp Kit on the Illumina HiSeq 2500 or Novaseq 6000 platform.

BUFFER LIST

lysis buffer

10 mM Tris-HCl (pH 8.0)
10 mM NaCl
0.2% NP-40

DNase I digestion buffer I (per tube)

10x DNase I digestion buffer	5 µl
10 mM $MnCl_2$	5 µl
10% SDS	2 µl
H_2O	88 µl

DNase I digestion buffer II (per tube)

10x DNase I digestion buffer	5 µl
10 mM $MnCl_2$	5 µl
10% Triton X-100	20 µl
H_2O	70 µl

DNase I stop buffer

125 mM EDTA
2.5% SDS

ChIP dilution buffer

50 mM Tris-HCl (pH 8.0)
167 mM NaCl
1.1% Triton X-100
0.11% NaDOC

SDS lysis buffer

50 mM Tris-HCl (pH 8.0)
1% SDS
10 mM EDTA

RIPA 500mM buffer

500 mM NaCl
50 mM Tris-HCl (pH 8.0)
1 mM EDTA
0.1% SDS
1% Triton X-100
0.1% NaDOC

Proteinase K buffer

10 mM Tris-HCl (pH 7.5)
1% SDS
15 mM EDTA

1x WB buffer

5 mM Tris-HCl (pH 7.5)
0.5 mM EDTA
1 M NaCl
0.02% Tween-20
